# Successful Outcomes for Whom and for What? Comment on "Government Actions and Their Relation to Resilience in Healthcare During the COVID-19 Pandemic in New South Wales, Australia and Ontario, Canada"

**DOI:** 10.34172/ijhpm.2022.6944

**Published:** 2022-04-25

**Authors:** Karina Aase

**Affiliations:** Centre for Resilience in Healthcare, Faculty of Health Sciences, University of Stavanger, Stavanger, Norway.

**Keywords:** COVID-19, Outcomes, Resilience in Healthcare, Stakeholders, Norway

## Abstract

The coronavirus disease 2019 (COVID-19) pandemic is the case of a complex global crisis that requires resilient performance across all levels of healthcare systems worldwide. The analysis of government actions during the COVID-19 pandemic in Australia and Canada by Smaggus et al raises the possibility for a much needed and essential debate on the notion of outcomes in the resilience in healthcare literature. Government actions have downstream effects on stakeholders’ capacity for resilience throughout the healthcare system. Yet, outcomes are fluent, multi-faceted and dependant on time, space, and stakeholder perspectives. It is appropriate and timely for the resilience in healthcare field to better grasp the nuances of successful outcomes in complex adaptive systems.

## Introduction

 In their article, Smaggus and colleagues^1^ give an exemplary account of government actions during the coronavirus disease 2019 (COVID-19) pandemic in the state of New South Wales Australia and in the province of Ontario Canada. The innovative aspect of their analysis is that it relates the government actions to the concept of resilience. This is especially important as there is a dearth of literature concerning macro level structures and actions and their role in resilience in healthcare.^2^

 Smaggus et al^1^ conceptualise resilience through the four potentials of responding, monitoring, anticipating, and learning, and methodologically base their study on an analysis of media releases from the two governments. The authors did not aim for a direct comparison of outcomes across the jurisdictions yet wanted to identify high-level themes regarding resilience in healthcare. A comparison of outcomes would probably not be feasible or even useful given the methodological approach chosen and the contextual complexity inherent in the two health system settings. Yet, it could be claimed that a better nuancing of outcomes as part of the understanding of resilience in healthcare would benefit the authors’ approach and the research field in general. The lack of emphasis on outcomes in the current literature is confirmed by a recent systematic review of the published literature on resilience in healthcare.^3^ Out of thirty-six studies only five included outcomes as part of their conceptualisation of resilience.

## What Are Outcomes in a Resilience Perspective?

 A basic foundation for the resilience perspective is the emphasis on positive, successful outcomes of healthcare processes. Expected or acceptable outcomes are other notions commonly used. Successful outcomes are the result of work processes where things go right, which should be seen as the normal or usual state of the system. Unusual or negative outcomes are when things go wrong, for example resulting in adverse events. The core concept of the resilience perspective is the value in learning from the full range of work outcomes, successful and unsuccessful, not concentrating entirely on preventing negative outcomes.^3^ Despite inevitable risks and complexity in healthcare processes, the system and its stakeholders adjust performance to uphold the frequency of successful outcomes. As such, outcomes are emerging from variability due to everyday adjustments rather than being a result of common cause-effect chains.^4^

 In the published literature on resilience in healthcare examples of types or emergence of outcomes are rare. Useful exceptions are McCray et al^5^ linking the performance of integrated teams in the health and social care sector to service user outcomes, Raben et al^6^ describing the emergent properties of the outcomes of early detection of sepsis in a medical ward, and Laugaland et al^7^ describing the performance variability involved in the hospital discharge outcomes of older patients.

## A Study of Stakeholder Perspectives on the Outcomes of Government Actions

 In 2015, we conducted a study on government actions to improve coordinated care across specialist and primary healthcare in Norway (the Coordination Reform) using the resilience perspective as a backdrop with a particular focus on outcomes.^8^ Based on the literature, resilience was defined as the ability of a healthcare system to succeed under varying conditions to increase the proportion of intended and acceptable outcomes.^9^

 The Norwegian Coordination reform was originally set out to improve the patient flow between hospitals and primary care institutions and to overcome challenges with delayed discharge better known as ‘bed blocking’ (ie, patients blocking beds in specialist care while awaiting municipal services). Financial measures were implemented to facilitate rapid discharge involving municipal co-financing of the specialist healthcare services including financial responsibility for patients ready for discharge. The study involved patients and their carers, specialist healthcare professionals and primary care professionals, and found that the three stakeholders viewed the outcomes of the reform quite differently. Taken from a hospital perspective, outcomes imposed by the reform were perceived mainly as successful. Taken from a primary care perspective, the picture was more nuanced and outcomes were perceived as variable and sometimes problematic. The patient and carer perspective adds further complexity to the comprehension of outcome as it reported mainly negative outcomes in the forms of poor involvement, unpreparedness, insecurity, stress, and the physical and mental challenges induced by an increase in the number of care transitions post-discharge.

 In sum, the downstream effects of the government actions to improve continuity of care could be deemed successful from one perspective but not from the viewpoint of others. This might also be the case for upstream effects of adjustments made at the local level of the healthcare system. What is valued as a successful outcome of a resilient performance at a practice level is not necessarily valued as positive at a higher level as the practice adjustment is then seen in relation to other practices of the system. In fact, local resilient performance might induce vulnerability in the system as a whole.^10^

## Outcomes in the Governance of the COVID-19 Pandemic

 The governance of the COVID-19 pandemic makes a highly interesting case for studying the fluent and emergent properties of outcomes in a complex adaptive healthcare system. Smaggus et al^1^ find that government actions in the face of the pandemic are characterised by monitoring and responding, while learning and anticipation are less pronounced. The authors conclude that the official communications issued by the governments of New South Wales and Ontario focused on the reactive aspects of resilience. This might also have consequences for the relative weight the governments put on outcomes where direct and short-term outcomes could potentially be prioritised.

 With a high degree of uncertainty during the COVID-19 pandemic, government actions may have intended and unintended outcomes, acceptable or unacceptable. Outcomes in a large-scale crisis is as such an emergent phenomenon that furthermore will change over time and space. In the early phases of the pandemic mortality, frequency of infected citizens, and availability of hospital beds and ventilators might constitute the essential outcomes. In later phases mental health of adolescents and older persons, or staff burnout might be vital to include as the basis for government actions. Outcomes of a global pandemic will also entail spatial differences where for example healthcare staff capacity differs across countries. An integrative review documents that data from the United States showed a decrease in staff resilience, whereas participants from China had increased resilience compared with pre-pandemic levels.^11^

 Smaggus et al^1^ discuss important challenges for learning and anticipation in the governments’ COVID-19 actions due to the novelty of possible threats, the degree of uncertainty, the level of interconnections and interdependencies, and the need to change course if chosen measures or guidance proved counterproductive. These same issues have direct consequences for the understanding of outcomes in the context of a pandemic. In this lies the recognition that specific outcomes in a complex crisis are rarely final endpoints. Most outcomes are themselves positive and/or negative influences contributing to subsequent outcomes and stages. In this process, learning and reflecting is crucial, while time to do so is a limited resource. The knowledge base is constantly insufficient, posing challenges for the anticipation of the complex web of short-term and long-term, direct and indirect outcomes. As Smaggus and colleagues’^1^ analysis is conducted in a period in which the pandemic was in a midway phase (December 2019 to August 2020) it would be important to continue the analysis to see whether the resilience potentials of anticipation and learning were more pronounced in governments’ actions as their knowledge bases increase.

## Implications

 The COVID-19 pandemic case as analysed by Smaggus et al^1^ and further discussed here accentuates the view of outcomes as an emergent phenomenon dependant on the resilience potentials of learning and anticipation. In the resilience in healthcare literature there is a need to clarify the notion of acceptable, successful outcomes, for whom and for what. Different outcomes represent different judgement of values that need to be explored and acknowledged in order to be able to share a common ground on what constitutes outcomes. Researchers and healthcare systems need to be able to differentiate types of outcomes related to different stakeholder groups, and to characterise the emergent properties of outcomes across time and space. Such development should also better integrate outcomes in a clinical and health services perspective with the broader outcomes in a public health and population level perspective.

## Ethical issues

 Not applicable.

## Competing interests

 Author declares that she has no competing interests.

## Author’s contribution

 KA is the single author of the paper.

## References

[1] Smaggus A, Long JC, Ellis LA, Clay-Williams R, Braithwaite J (2022). Government actions and their relation to resilience in healthcare during the COVID-19 pandemic in New South Wales, Australia and Ontario, Canada. Int J Health Policy Manag.

[2] Berg SH, Akerjordet K, Ekstedt M, Aase K (2018). Methodological strategies in resilient health care studies: an integrative review. Saf Sci.

[3] Iflaifel M, Lim RH, Ryan K, Crowley C (2020). Resilient health care: a systematic review of conceptualisations, study methods and factors that develop resilience. BMC Health Serv Res.

[4] Hollnagel E. FRAM: The Functional Resonance Analysis Method: Modelling Complex Socio-Technical Systems. Farnham, Surrey: Ashgate Publishing; 2012.

[5] McCray J, Palmer A, Chmiel N (2016). Building resilience in health and social care teams. Pers Rev.

[6] Raben DC, Viskum B, Mikkelsen KL, Hounsgaard J, Bogh SB, Hollnagel E (2018). Application of a non-linear model to understand healthcare processes: using the functional resonance analysis method on a case study of the early detection of sepsis. Reliab Eng Syst Saf.

[7] Laugaland K, Aase K, Waring J (2014). Hospital discharge of the elderly--an observational case study of functions, variability and performance-shaping factors. BMC Health Serv Res.

[8] Laugaland K, Aase K. The demands imposed by a health care reform on clinical work in transitional care of the elderly: a multi-faceted Janus. In: Hollnagel E, Braithwaite J, Wears RL, eds. Resilient Health Care: The Resilience of Everyday Clinical Work. Vol 2. Farnham, Surrey: Ashgate Publishing; 2015.

[9] Hollnagel E, Braithwaite J, Wears RL. Resilient Health Care. Farnham, Surrey: Ashgate Publishing; 2013.

[10] Berg SH, Aase K (2019). Resilient characteristics as described in empirical studies on health care.

[11] Baskin RG, Bartlett R (2021). Healthcare worker resilience during the COVID-19 pandemic: an integrative review. J Nurs Manag.

